# Perspectives on Telemedicine during the Era of COVID-19; What Can Saudi Arabia Do?

**DOI:** 10.3390/ijerph182010617

**Published:** 2021-10-11

**Authors:** Ali Mohsen Al-Hazmi, Haytham A. Sheerah, Ahmed Arafa

**Affiliations:** 1 Health Promotion and Health Education Research Chair, College of Medicine, King Saud University, Riyadh 11461, Saudi Arabia; aalhazmii@ksu.edu.sa (A.M.A.-H.); ahmed011172@med.bsu.edu.eg (A.A.); 2 Department of Family & Community Medicine, College of Medicine, King Saud University, Riyadh 11461, Saudi Arabia; 3 Department of Preventive Cardiology, National Cerebral and Cardiovascular Center, Suita 564-8565, Japan; 4 Public Health, Department of Social Medicine, Graduate School of Medicine, Osaka University, Osaka 565-0871, Japan; 5 Department of Public Health, Faculty of Medicine, Beni-Suef University, Beni-Suef 62511, Egypt

**Keywords:** COVID-19, telemedicine, data security, medical practice, Saudi Arabia

## Abstract

The coronavirus disease 2019 (COVID-19) pandemic has represented a challenge to medical practice in Saudi Arabia and worldwide. In contrary to the increasing numbers of COVID-19 patients, there was a limitation in the capacity of medical practices and access to healthcare. A growing body of evidence from healthcare settings in Saudi Arabia and worldwide has suggested a possible role for telemedicine in responding to this evolving need. Telemedicine can be used for triage, direct care, follow-up, and consultation. It could be delivered through synchronous, asynchronous, and mixed approaches. While telemedicine has several advantages, such as accessibility and cost-effectiveness, its diagnostic reliability should be further investigated. The Saudi Vision (2030) has drawn up a roadmap to invest in digital healthcare during the coming decade; however, some barriers related to using telemedicine in Saudi healthcare settings, including cultural issues and technical difficulties, should be openly discussed. In addition, before putting telemedicine in practice on a wide scale in Saudi Arabia, more efforts should be carried out to issue updated legislation and regulations, discuss and respond to ethical concerns, and ensure data security.

## 1. Introduction

After spreading to more than 110 countries, the novel coronavirus disease (COVID-19) was declared a pandemic on 11 March 2020 [1]. With the increasing numbers of morbidities and mortalities, most countries had to take strict actions to prevent the spread of COVID-19, such as imposing lockdown and suspending travel [2]. The new situation has represented a challenge to medical practice. Regular check-ups, elective surgeries, and follow-ups were discouraged, and healthcare settings became potential sources of COVID-19 infection [3,4]. Healthcare workers had to face waves of COVID-19 cases, significant shortfalls in personal protective equipment, a lack of social and institutional support, working extra hours, role conflict and ambiguity attributed to the changing management protocols and incompetent training, and exposure to workplace violence [5,6,7]. Further, many healthcare workers had to self-isolate after getting in contact or infected with COVID-19, which led to shortages in medical staff and undermined the quality of healthcare [5]. These factors together reduced the capacity of medical practice and restricted people’s access to healthcare [5]. 

Saudi Arabia was among the most affected countries by the COVID-19 pandemic in the Eastern Mediterranean Region, with a total of 537,374 confirmed patients and 8388 related deaths by 14 August 2021 [8]. As a consequence, the Saudi government took decisive measures to prevent the spread of COVID-19, such as imposing lockdown, enforcing social distancing measures, suspending public transportation, schools, and universities, preventing religious mass gatherings, and tracking travelers with possible COVID-19 infections. Moreover, it worked on improving the capacity of early detection and management of COVID-19 cases via preparing several primary healthcare centers with personnel and equipment to receive people with COVID-19 symptoms and enhancing the preventive capacity by providing the COVID-19 vaccine on a wide scale [8]. These regulations made face-to-face medical consultation even harder. Like most countries worldwide, the increasing need for healthcare in Saudi Arabia during the COVID-19 pandemic was faced with a diminished capacity of medical practice and limited access to healthcare [8,9].

The evolution of the COVID-19 pandemic and the associating restrictive measures have raised the awareness of telemedicine and resulted in a rapid increase in the volume of the provided telemedicine services. To fill this gap, several digital solutions have been suggested. With the dramatic improvement in telecommunication technologies, telemedicine has become one of these solutions that can allow healthcare workers to interactively communicate with distant patients to provide them with diagnostic and therapeutic services [9,10,11,12,13,14]. In this context, this review article aims to discuss the uses, delivery approaches, and characteristics of telemedicine and highlight the potential benefits of telemedicine for the Saudi healthcare system.

## 2. Methods

To draft this review, we searched PubMed for articles that discussed the potential uses of telemedicine during the COVID-19 pandemic using the search terms “Telemedicine” and “COVID-19”. We also checked the reference sections of the reviewed articles to obtain related studies. 

## 3. Uses of Telemedicine 

Telemedicine has four primary uses: triage, direct care, follow-up, and consultation [11,12,13,14]. In triage, healthcare workers decide on the priority of the patient’s health condition for treatment or referral [13]. The COVID-19 pandemic has resulted in unprecedented demand on intensive care unit services. The use of telehealth for triaging cases could be a useful way of assessing patients and determining the most appropriate clinical care [14]. 

In direct care, healthcare workers, typically physicians, consult their patients by a video meeting, and patients may send their labs and radiology to their physicians. Patients can be screened for possible symptoms of COVID-19 and receive medical advice [13,14]. Besides, since the prevalence of psychiatric disorders has been increasing among the public and healthcare workers as a result of the COVID-19 pandemic and its consequences [15,16,17,18], telemedicine can be used to provide psychiatric counseling [13].

In follow-up, physicians monitor the prognosis of health conditions and their response to medications. This is particularly important for patients with chronic health conditions and people seeking weight management and nutritional services [13]. Patients isolated at home for having COVID-19 can make benefit from telemedicine by communicating with physicians who can monitor the prognosis of their symptoms online and change the management plan accordingly [14].

In consultation, primary care or junior physicians seek advice from specialized or senior physicians regarding their patients’ diagnosis and management plans. Thus, telemedicine can be used for training and educative purposes, especially for junior healthcare workers and those assigned in remote areas [12,13,14].

## 4. Delivery of Telemedicine 

Telemedicine services can be delivered via three major approaches: synchronous, asynchronous, and mixed. In the synchronous approach, also known as the real-time approach, patients directly communicate with their physicians via video meetings. This approach is quite similar to face-to-face consultation [11,14]. In the asynchronous approach, also known as the store-and-forward approach, patients send photos of their lesions, scans of their radiology and lab findings, and a summary of their complaints to their physicians who respond using the same manner [11,14]. The pros and cons of the synchronous and asynchronous approaches were summarized (Table 1). 

In the mixed approach, also known as the hybrid approach, patients first send their labs and radiology like the asynchronous approach before having an online video meeting or a phone call with their physicians like the synchronous approach [11,14]. This method can get over most of the disadvantages of the synchronous and asynchronous approaches.

## 5. Examples of Telemedicine during the COVID-19 Pandemic Worldwide

In several healthcare settings worldwide, telemedicine was used to efficiently provide medical services during the COVID-19 pandemic. For example, the University of California, San Diego Health, developed electronic health record-based tools to provide medical care [19]. This electronic system included video meetings with patients, triage of patients via phone calls, giving home isolation instructions for COVID-19 patients, tracking COVID-19-related infections, screening and treating urgent cases, and providing decision support for patients and junior physicians [19]. 

In China, a study conducted on 161 tertiary hospitals representing 29 provinces showed that 93.8% of tertiary hospitals provided synchronous and asynchronous telemedicine services during the COVID-19 pandemic and 75.8% of hospitals had assigned telemedicine staff [20]. Another study conducted on 48 public dental hospitals in China during the COVID-19 pandemic showed that 90% of hospitals changed their face-to-face consultations to web-based and mobile-based consultations, and telemedicine triage to detect the cases that needed urgent intervention was carried out in 69% of the included hospitals [21]. 

A survey of ophthalmologists working in India showed that 72.5% of respondents stopped seeing patients due to the COVID-19 lockdown, and 82.9% of those who continued seeing patients confined their consultation to emergency cases. However, telemedicine was able to mitigate this difficult situation as 77.5% of ophthalmologists said that they conducted telephone and video consultations during the lockdown using different telemedicine applications and social network sites [22].

A team of local experts in Malta developed a telemedicine hub that included data about the newly diagnosed COVID-19 patients and coordinated medical assessment and management for those patients. During the early months of the COVID-19 pandemic, this hub safely managed 91% of COVID-19 patients in the country [23]. The University Hospital of Zurich implemented medical chatbots to offer early diagnosis, treatment, and medical information for remotely situated patients with dermatological conditions [24]. In Italy, most hospitals and clinics had to cancel their clinic appointments, reduce inpatient consultations, and postpone nonurgent surgeries due to COVID-19 restrictions [25].

Yet, telemedicine has flourished during the same period, with many hospitals and healthcare workers resorted to online consultations to manage patients with urological [26], dermatological [27], hepatic [28], immunological [29], and endocrinal conditions [30]. 

## 6. Examples of Telemedicine during the COVID-19 Pandemic in Saudi Arabia

Saudi Arabia was not far from putting telemedicine in practice during the COVID-19 pandemic. The Ministry of Health in Saudi Arabia and the private sector had developed several telemedicine services before and during the COVID-19 pandemic as a part of the Saudi Vision (2030) that managed to invest in digital health and provide innovative digital solutions for the increasing need for healthcare [9,31,32]. These digital healthcare services have attracted more than two million users and were efficiently incorporated into healthcare services in the country [31]. Some of these applications (*Sehha*, *Cura*, *MayaClinic*, *Nala*, and *Labayh*) provided telemedicine services either synchronously via video meetings or asynchronously via text messages and chatbots [32]. The new digital healthcare services have been used to curb the COVID-19 pandemic in Saudi Arabia. For example, individuals with possible COVID-19 symptoms in Saudi Arabia can use *Sehaty* and *Mawaid* platforms and applications to check their health status as a form of digital screening [32]. Confirmed COVID-19 cases can report their epidemiological data and contact details using the *Taqasi* application as a form of surveillance. They can also receive medical advice and follow-up consultation from healthcare workers via the *Tetamman* application [32].

During the COVID-19 pandemic, an online cross-sectional survey conducted on 392 physicians in Saudi Arabia (51.5% consultants, 29.3% specialists, and 19.2% residents) showed that 58.1% of them used telemedicine via *WhatsApp* (53.8%), *Zoom* (33.4%), e-mails (21.4%), the *Sehha* application (16.5%), and *Microsoft Teams* (6.2%) [33]. Slightly less than half of the physicians reported that their healthcare settings were equipped with telemedicine facilities. When physicians were asked about their perceptions of telemedicine, 89.5% reported that its effectiveness depended on the specialty, 88.5% that it could be better used for monitoring patients with chronic conditions, 87.5% that it reduced unnecessary visits, 63.5% that it was cost-effective, 61% that patients were satisfied with the online consultation, and 27.8% reported a good diagnostic concordance with the face-to-face consultations [33]. The barriers to applying telemedicine, according to the physicians, were as follows: technological limitations (66.6%), concerns about diagnostic reliability (66.1%), cultural issues (53.3%), and physician resistance (36.5%) [33].

A limited-scale study including 25 physicians in two hospitals in Taif, Saudi Arabia, showed that 16 of them had fair to good knowledge about telemedicine [34]. Although only 19 physicians reported that their hospitals provided information and training on telemedicine, all participating physicians have used sorts of telemedicine services during the COVID-19 pandemic (14 telephone, 12 video, 11 social media, 4 text messages, and 3 e-mails) [34]. More than a third of physicians praised the advantages of telemedicine in the form of reducing time, saving money, and enhancing the quality of healthcare. Most physicians held a belief that telemedicine services should be continued in the Saudi healthcare settings after the COVID-19 pandemic [34]. 

In addition to exploring the readiness of healthcare workers in Saudi Arabia to provide telemedicine services, it was also important to consider the opinions of patients about these new forms of healthcare services. One study used an online survey to assess the satisfaction of 425 patients treated by telemedicine in Saudi Arabia during the early months of the COVID-19 pandemic [35]. Of the participants, 83.8% were satisfied with the ease of registration and scheduling, 80.3% with the ability to understand recommendations, 80.2% with the ability to talk freely, 74.8% with the quality of healthcare provided, 78.1% with the quality of visual image, and 78.1% with the quality of audio sound. Overall, 77.9% were satisfied with the telemedicine experience [35]. Despite 84.9% of participants reported that telemedicine made access to healthcare easier, 51.1% said that they would prefer face-to-face over telemedicine consultation and 59.8% said that they were not willing to take part in a telemedicine consultation again. The main concern of most participants (80.2%) was that the presence of a camera and other telemedicine equipment made them uncomfortable [35].

Another study assessed the satisfaction of 41 patients who had rhinoplasty and were receiving telemedicine follow-up services during the time of COVID-19 in Saudi Arabia [36]. All participants were satisfied with the technical issues (easy registration, clear voice, and no disconnection). More than half of the participants were confident that their physicians could assess their medical condition and evaluate their medication requirements using telemedicine. Only seven patients reported inability to explain their medical problem to their physicians and four patients felt uncomfortable with telemedicine equipment [36].

One study investigated the access to healthcare during the COVID-19 pandemic among users and nonusers of the *Sehha* application developed by the Ministry of Health in Saudi Arabia in 2018 to provide telemedicine services [37]. Users of the *Sehha* application had a significantly higher score of access to healthcare, satisfaction with the provided healthcare, and efficiency than nonusers [37]. However, some technical issues were reported by the users of the applications. Furthermore, there were significant differences between users and nonusers regarding their age, sex, education, and region [37]. 

In a prospective pre-/post interventional study, 130 patients with type II diabetes who were living in Saudi Arabia during the COVID-19 pandemic received telemedicine care [38]. After four months, their HbA1c significantly improved from 9.98 ± 1.33 pre-telemedicine to 8.32 ± 1.31 post-telemedicine. Most patients needed only one or two face-to-face consultations throughout the four months of intervention instead of the regular weekly face-to-face consultation [38]. 

It should be noted that there were a few national studies that assessed knowledge and perceptions about telemedicine before the time of the COVID-19 pandemic, and those studies showed, compared with the post-COVID-19 studies, inadequate knowledge and negative attitudes towards telemedicine in Saudi Arabia [39,40,41,42,43]. The improving knowledge and attitudes towards telemedicine during the post-COVID-19 studies are good indicators for implementing telemedicine on a wide scale in the country.

## 7. Issues to Be Considered before Implementing Telemedicine in Saudi Arabia

Before applying telemedicine on a wide scale in Saudi Arabia, a few questions should be answered.

- Is telemedicine accessible?
- Is telemedicine accurate?
- Is telemedicine safe?
- Is telemedicine cost-effective?
- Are there any ethical considerations related to telemedicine?
- How can we secure telemedicine data?

Unfortunately, clear-cut answers to these questions cannot be obtained from studies conducted in Saudi Arabia because of their limited number, however, research evidence from other countries could help us speculate answers that may aid in applying telemedicine on a wider scale in the country.

Thanks to telemedicine, several patients with chronic diseases were able to avoid exposure to the COVID-19 infection in healthcare settings and receive their diagnosis, treatment, and monitoring at home. Although telemedicine can offer a wider coverage of healthcare, individuals who are not familiar with technologies, those who do not have broadband fast internet, illiterate people, older adults, and people with specific disabilities such as hearing loss and blindness may find difficulties in using telemedicine services [44,45]. In Saudi Arabia, a study assessing the use of the *Sehha* application that provides telemedicine services in the country, revealed that older adults, women, and people living in regions with inadequate internet services were less likely to use the application [37]. Facing technical problems was the major concern of nonusers and unsatisfied users [37]. Therefore, the issue of telemedicine accessibility should be well-addressed before investing in telemedicine on a broad scale in Saudi Arabia. Developers of telemedicine applications can use some techniques to make the applications easier to use, such as labeling options with descriptive photos, reducing the steps to obtain the healthcare service, offering voice explanations, and regularly working on the evolving technical problems.

One of the issues that should be discussed is the accuracy of telemedicine. A recent study showed that two-thirds of physicians in Saudi Arabia were concerned about the diagnostic reliability of telemedicine [33]. Yet, previous studies detected comparable accuracy between face-to-face diagnosis and telediagnosis in patients with skin cancers [46,47], diabetic retinopathy [48], burns [49,50], muscle tear [51], dementia [52], and uncontrolled hypertension [53]. However, it should be noted that this accuracy is most probably related to the quality of video and audio used in the online consultation, availability of updated equipment in healthcare settings, and availability of healthcare workers with telemedicine experience. Previous studies from Saudi Arabia revealed that the quality of audio and video in most online consultations was satisfactory, yet some technical issues evolved from time to time [33,34,35,36,37]. Still, the availability of telemedicine equipment in Saudi healthcare settings and providing healthcare workers with the required training to use telemedicine remain a challenge. 

Further, telemedicine was shown to be a safe approach. One meta-analysis showed that telemedicine could safely help with glucose monitoring in diabetic patients, following up patients with hypertension, guiding junior physicians at neonatal intensive care and emergency departments, and triaging patients in neurosurgery [54].

Financially, compared with face-to-face consultation, telemedicine is considered cost-effective in several ways. It cuts transportation time, reduces inpatient visits and referrals to secondary and tertiary healthcare settings, decreases lost productivity, saves costs of companions in the case of children and patients with disabilities, and avoids drawbacks related to delayed diagnosis [55,56]. A previous study conducted on physicians from Saudi Arabia showed that almost two-thirds of respondents held a belief that telemedicine during the COVID-19 pandemic was cost-effective, however, cost-effectiveness in that study was subjectively assessed [33]. Thus, large studies to investigate the cost-effectiveness of telemedicine in public and private healthcare settings in Saudi Arabia are highly warranted.

Obtaining informed consent before the traditional clinical examination or telemedicine is mandatory. Since telemedicine has specific features, the patient should understand the policies regulating the use of webcams, saving conversations and photos, and sharing health data with other healthcare workers [57]. Signing informed consent in telemedicine can be needed in specific situations either by signing a paper informed consent before scanning it and sending it to the healthcare worker or simply signing an electronic form. In both situations, a detailed explanation, either online or video-recorded, is needed to describe the steps of telemedicine [57,58]. Therefore, the authorities in Saudi Arabia should work on updating the laws that regulate telemedicine practice, developing clear legislations about how patients can give their informed consent, deciding on the situations where healthcare workers should save or delete photos and conversations of telemedicine consultation, and deciding on the situations where general practitioners or junior physicians may need to share medical data including photos with specialized or senior physicians as a second opinion. 

Data security is a major concern of many telemedicine users [56,57]. In a country with a conservative culture, such as that of Saudi Arabia, people, especially women, may not feel comfortable with sharing their medical history or showing their bodies through cameras. They might also abstain from sending photos showing their lesions or pathologies for the same reason [35]. Therefore, personal data leakage or sharing data and photos of patients with unauthorized parties can ruin the future of telemedicine in the country. Some techniques should be adopted to improve data security, such as replacing patients’ identifying data with codes, entity authentication, limiting access to authorized personnel, encrypting data using usernames and complicated passwords, avoiding sending photos to the personal devices of healthcare workers, regular scanning for viruses, and filtering electronic feeds [59].

## 8. Conclusions

Telemedicine can offer a convenient way of expanding access to healthcare in Saudi Arabia accurately and cost-effectively while minimizing the risk of COVID-19 transmission. More efforts should be exerted to provide healthcare settings with technical equipment and training needed for telemedicine. Regulations to implement telemedicine on a large scale in Saudi Arabia while protecting data privacy are also needed. 

## Figures and Tables

**Table 1:** Pros and cons of the synchronous and asynchronous approaches of telemedicine delivery.

Delivery Approach	Pros	Cons
Synchronous	Realistic Timely fashion Like face-to-face consultation	Requires previous coordination Requires high-speed internet Video quality can be inadequate Patients feel shy to show their bodies on a webcam More expensive than the asynchronous approach May interrupt workflow
Asynchronous	Flexible Does not require a high-speed internet Images usually have good quality Cheap Does not interrupt workflow	Physicians cannot ask for unclear information Requires adequate storage space

## Data Availability

Not applicable.
